# Supplementary material for: Multiple Na,K-ATPase Subunits Colocalize in the Brush Border of Mouse Choroid Plexus Epithelial Cells
Source: Int J Mol Sci. 2021 Feb 4;22(4):1569. doi: 10.3390/ijms22041569 (PMC7915972; doi:10.3390/ijms22041569)
Supplement: Supplementary file 1 [file ijms-22-01569-s001.pdf]

Figure S1: Migration of CP protein homogenates in native PAGE. A) Representative gel showing CP proteins separated by native PAGE. Arrowheads mark distinct bands. Molecular sizes are shown on the left (kDa). B) Immunoblot for Na,K-ATPase α1 (NaK α1) of a similar gel run in parallel. Arrowhead mark the immunoreactive band. C) The same gel as in panel A) after excision of the 3 bands for protein mass spectrometry. Arrowheads mark distinct bands. D) Co-migration of Na,K-ATPase α1 with other CPE proteins. Dual immunofluorescence blot after native PAGE with antibodies against Na,K-ATPase α1 (red) and NKCC1, Ankyrin-3, Na,K-ATPase α2, AE2, or NCBE (all in green), as indicated.

Figure S2: Super resolution colocalization of Na,K-ATPase subunits and AQP1 in the brush border of CPECs. Sections from mouse CP was immunostained for stimulated emission depletion (STED) microscopy. A) Dual labelling microscopy for Na,K-ATPase α1 (NaK α1, green) and β1 subunits (NaK β1, red). Yellow colour indicates colocalization within 30 nm. B) Similar labelling for the colocalization of Na,K-ATPase α1 (NaK α1, green) and Na,K-ATPase α2 (NaK α2, red) subunits. C) Positive control assay with two antibodies against the Na,K-ATPase β1 subunits (green and red, respectively). D) Labelling for the colocalization of Na,K-ATPase α1 (NaK α1, green) and aquaporin 1 (AQP1, red). Asterisks indicates CPEC border/tight junction area, and indicates the width of the CPEC which is approximately 10 μm. CY - CPECs cytosol, MV - microvilli, VL is the fourth ventricle lumen.

Figure S3: Control of Na,K-ATPase and NKCC1 PLA. A) Immunofluorescence micrograph of PLA products (red) with only anti-Na,K-ATPase α1 antibody (NaK α1) using secondary anti-mouse "+" DNA strand and "-" DNA strand antibodies with cell nucleus counterstain (blue) at low magnification. B) Higher magnification micrograph of the same structure overlaid on the corresponding DIC image. C) Bar graph representing the count of PLA reactions in the

indicated image areas. D) Immunofluorescence micrograph of PLA products (red) with the anti-Na,K-ATPase α1 antibody (NaK α1) using only secondary anti-mouse "+" DNA strand antibodies with cell nucleus counterstain (blue) at low magnification. E) Higher magnification micrograph of the same structure overlaid on the corresponding DIC image. F) Bar graph representing the count of PLA reactions in the indicated image areas. G) Immunofluorescence micrograph of PLA products (red) using only the anti-NKCC1 antibody using secondary anti-rabbit "+" DNA strand and "-" DNA strand antibodies with cell nucleus counterstain (blue) at low magnification. H) Higher magnification micrograph of the same structure overlaid on the corresponding DIC image. I) Bar graph representing the count of PLA reactions in the indicated image areas. Arrows indicate luminal surfaces, while arrowheads mark basolateral surfaces. VL denotes the fourth ventricle lumen.

Figure S4: Co-immunoprecipitation of Na,K-ATPase α1 with Na,K-ATPase β1 and Ankyrin-3 in the CP. Anti-Na,K-ATPase α1 and anti-Ankyrin-3 antibodies were used as bait for immunoprecipitation (IP) using either commercial elution buffer (Extraction buffer) or CHAPS buffer, as indicated below the blot. Anti-Proteasome 20S was used as control IP antibody. Molecular weights are indicated on the left (kDa), and the antibodies for immunoblotting (IB) are indicated right to the blots.

Figure S5: Control of Ncbe and NBCe2 PLA. A) Immunofluorescence micrograph of PLA products (red) with only the anti-Ncbe antibody using secondary anti-rabbit "+" DNA strand and "-" DNA strand antibodies with cell nucleus counterstain (blue) at low magnification. B) Higher magnification micrograph of the same structure overlaid on the corresponding DIC image. C) Bar graph representing the count of PLA reactions in the indicated image areas. Asterix indicates an estimated minimal number of reactions, as these were too confluent to

count in 2 of 3 mice. D) Immunofluorescence micrograph of PLA products (red) with only anti-NBCe2 antibody using secondary anti-rabbit "+" DNA strand and "-" DNA strand antibodies with cell nucleus counterstain (blue) at low magnification. E) Higher magnification micrograph of the same structure overlaid on the corresponding DIC image. F) Bar graph representing the count of PLA reactions in the indicated image areas. G) Immunofluorescence micrograph of PLA products (red) with the anti-NBCe2 antibody using only the secondary anti-rabbit "+" DNA strand antibody with cell nucleus counterstain (blue) at low magnification. H) Higher magnification micrograph of the same structure overlaid on the corresponding DIC image. I) Bar graph representing the count of PLA reactions in the indicated image areas. Arrows indicate luminal surfaces, while arrowheads mark basolateral surfaces. VL denotes the fourth ventricle lumen.

Figure S6: Comparison of the distribution of PLA reaction products. The PLA reaction counts in the lumen, the luminal membrane domain, the cytosol, the nucleus, the basal membrane domain, and the connective tissue are shown as % of all reactions counted for the indicated combination of primary antibodies (total numbers are indicated on the corresponding figures). All analyzed combinations with the antibodies against Na,K-ATPase α1 (NaK α1), Na,K-ATPase α2 (NaK α2), Na,K-ATPase β1 (NaK β1), NKCC1, AE2, Ncbe, NBCe2, β-actin (β-actin), α2-spectrin (α2 spec), and Ankyrin-3 (Ank3) are shown. The counts for the positive control reactions for NaK α1, NKCC1, and NBCe2 (applying a single primary antibody and both "+ probe" and "- probe" against that antibody) are also shown along the counts of negative control reactions for NaK α1, NKCC1 and NBCe2 (applying a single primary antibody and only the "+ probe" or "- probe" against that antibody).

Figure S7: RT-PCR analysis of spectrin expression in FACS isolated CPECs. Intact mouse choroid plexi were labelled with concanavalin A-fluorescein from the luminal side. The cells were separated and CPECs enriched by FACS to more than 99.9% purity. After reverse transcription, primers were applied with the cDNA in PCR reactions as indicated on the figure: Spectrin (Sp) forms (α1-2, β1-5). CPE designates FACS isolated CPECs, while Kid denotes the control tissues mouse kidney cortex. Molecular size is indicated left to the individual gels.

Figure S8: Super resolution co-localization of Na,K-ATPase subunits with actin and α2-spectrin in the brush border of CPECs. Sections from mouse CP was immunostained for stimulated emission depletion (STED) microscopy. A) Dual labelling microscopy for α2-spectrin (green) and Na,K-ATPase α2 subunits (NaK α2, red). Yellow color indicates colocalization within 30 nm. B) Similar labelling for the colocalization of β-actin (Actin, green) and Na,K-ATPase α1 subunits (NaK α1, red). Asterisks indicates CPEC border/tight junction area, and indicates the width of the CPEC which is approximately 10 μm. CY - CPECs cytosol, MV - microvilli, VL is the fourth ventricle lumen.

Figure S9: Transport protein abundances in various transgenic mouse models by immunofluorescence semi-quantitation. A) Abundance of Na,K-ATPase α1, -α2, -β1, AQP1, Ncbe and NBCe2 in NKCC1 wt (white bars) and NKCC1 ko (gray bars) mouse CPECs relative to NKCC1 wt (n=5). B) Abundance of Na,K-ATPase α1, -α2, -β1, and NKCC1 in AQP1 wt (white bars) and AQP1 ko (gray bars) mouse CPECs relative to AQP1 wt (n=5). C) Abundance of Na,K-ATPase α1, -α2, -β1, AQP1 and NKCC1 in NHE1 wt (white bars) and NHE1 ko (gray bars) mouse CPECs relative to NHE1 wt (n=5, vertical bars indicate statistical significance). D) Abundance of Na,K-ATPase α1, -α2, -β1, AQP1 and NKCC1 in NBCn1 wt (white bars) and NBCn1 ko (gray bars) mouse CPECs relative to NBCn1 wt (n=5). E) Abundance of Na,K-

ATPase α1, -α2, -β1, AQP1 and NKCC1 in homozygous Na,K-ATPase β2/β1 knock-in mouse (gray bars, n=7) CPECs relative to corresponding heterozygous mouse CPECs (hz, white bars, n=3).

Figure S10: Control cross-cellular immunolabeling profiles of mouse CPECs. A) Bar graph showing the average Na,K-ATPase β1 staining intensity (arbitrary units) for each of the 50 bins in CPECs from AQP1 wt (grey) and ko (black) mice (n = 5). B) Similar bar graphs showing the cross-cellular NBCe2 labeling profiles in CPECs from NKCC1 wt (grey) and ko (black) mice (n = 5). C) Bar graphs showing the cross-cellular Ncbe labeling profiles in CPECs from NKCC1 wt (grey) and ko (black) mice (n = 5). Bin #1 represents the basal end of the cell, while bin #50 is the luminal end. Individual observations are indicated as points, and error bars represent SEM.

Supplemental Table 1. P-values for plot-profile graphs with statistical significance in Figure 12 (Students two-tailed t-test).

**Bin**	**NaK a1**	**NaK a2**	**NaK b1**	**AQP1**
**2**		0.0229		
**3**	0.0216	0.0026		
**4**	0.0064	0.0009		
**5**	0.0020	0.0007		0.0271
**6**	0.0010	0.0001	0.0337	0.0073
**7**	0.0005	0.0002	0.0057	0.0012
**8**	0.0009	0.0000	0.0023	0.0010
**9**	0.0003	0.0000	0.0029	0.0012
**10**	0.0005	0.0000	0.0042	0.0027
**11**	0.0001	0.0000	0.0041	0.0303
**12**	0.0042	0.0009	0.0228	
**13**	0.0095	0.0108	0.0118	
**14**	0.0274	0.0035		

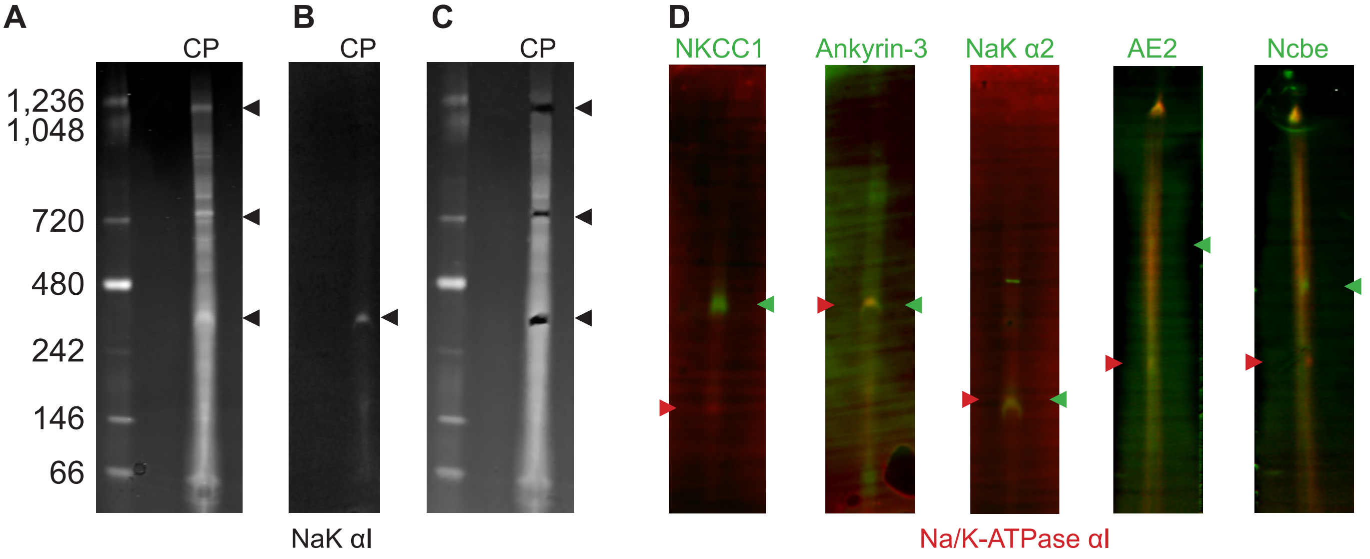

Supplemental Figure 1

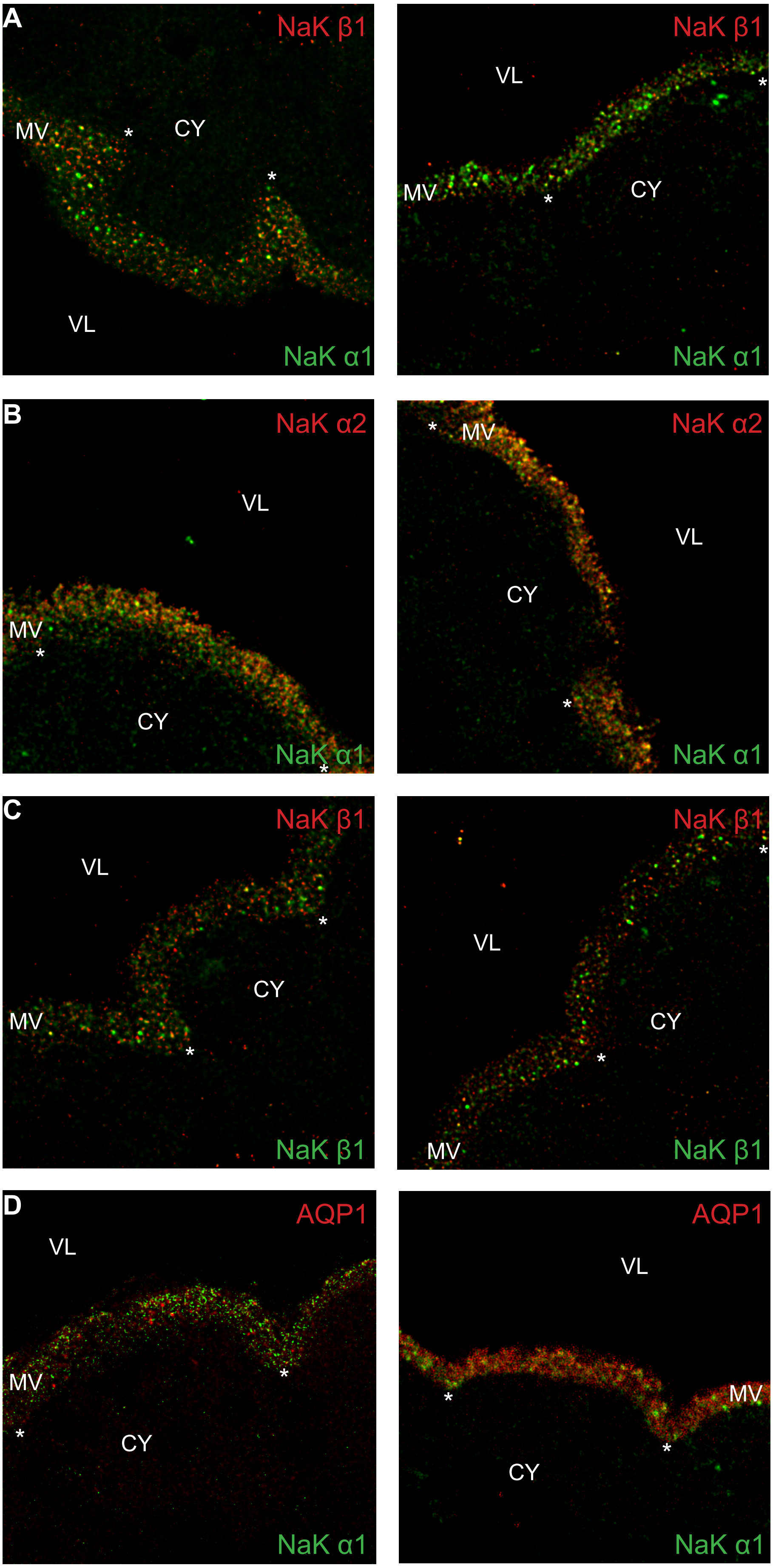

Supplemental Figure 2

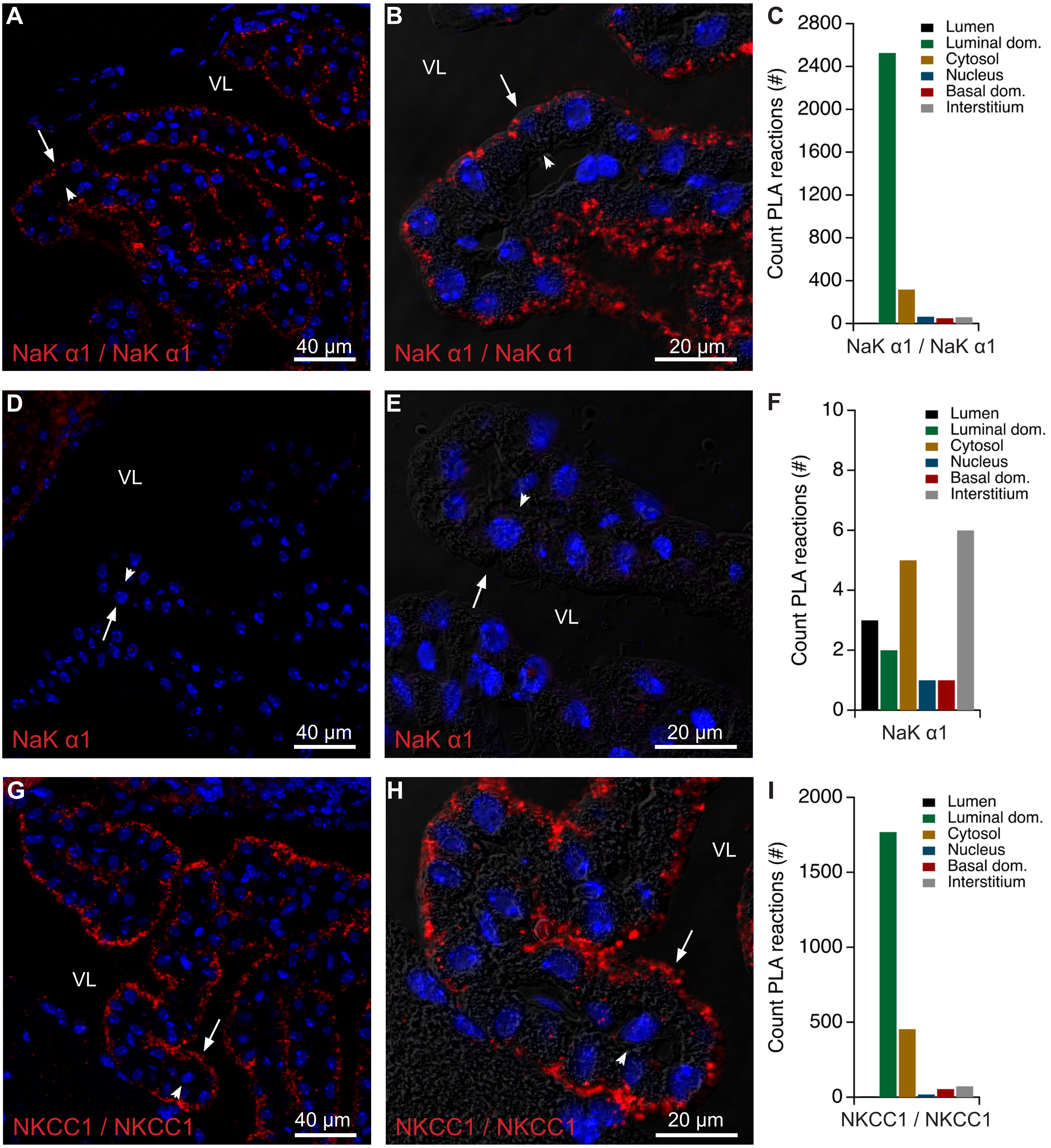

Supplemental Figure 3

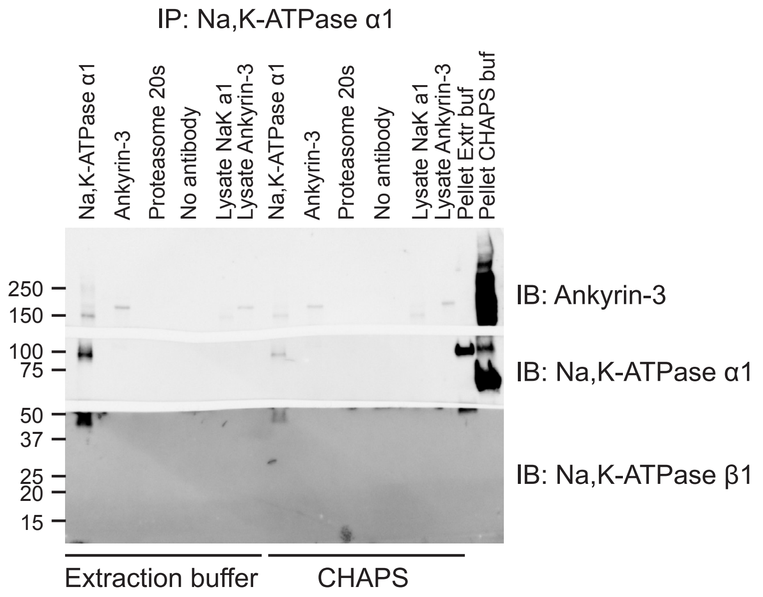

Supplemental Figure 4

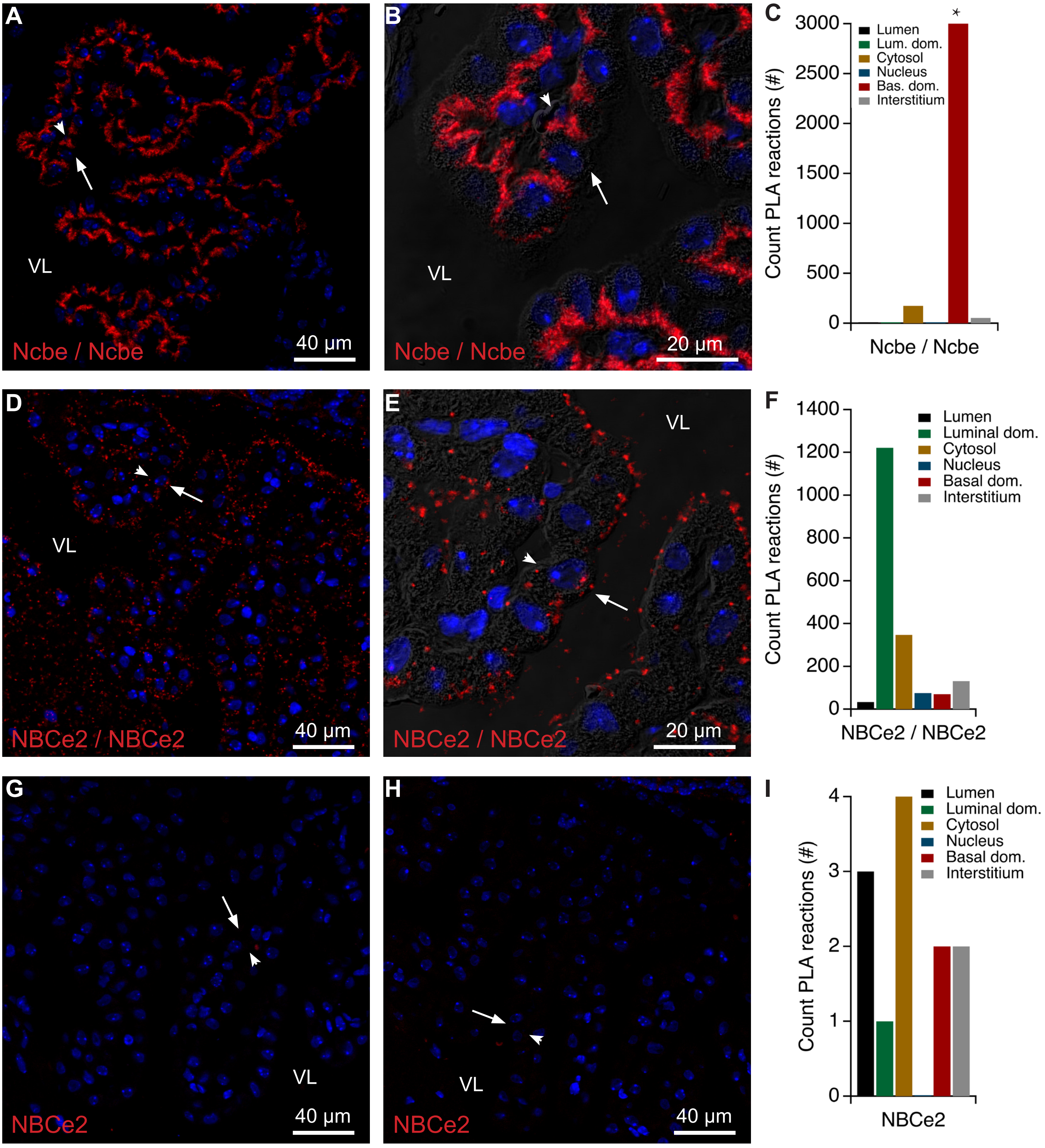

Supplemental Figure 5

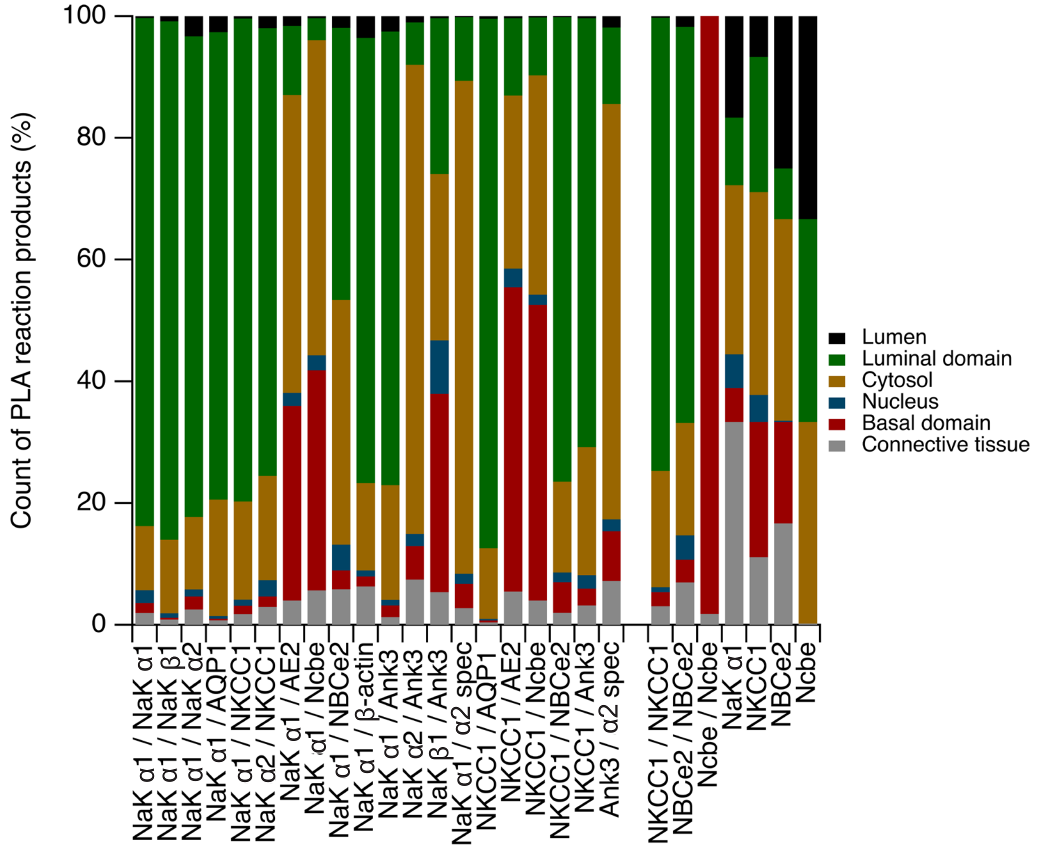

Supplemental Figure 6

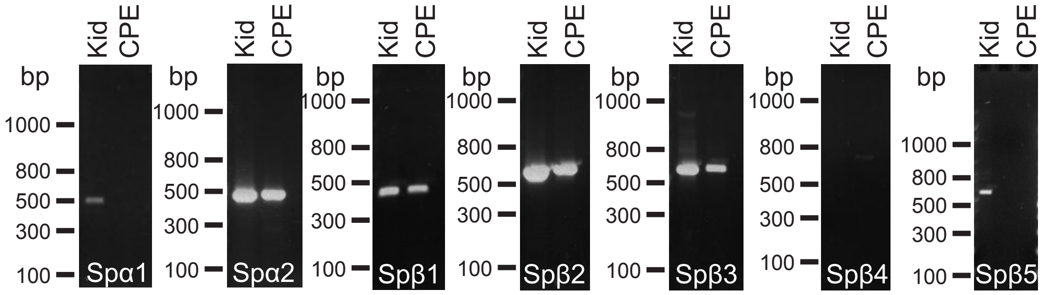

Supplemental Figure 7

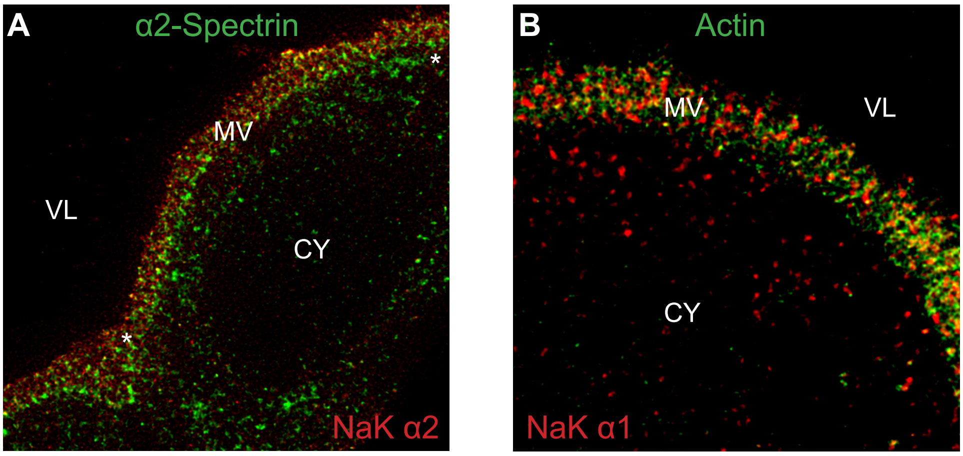

Supplemental Figure 8

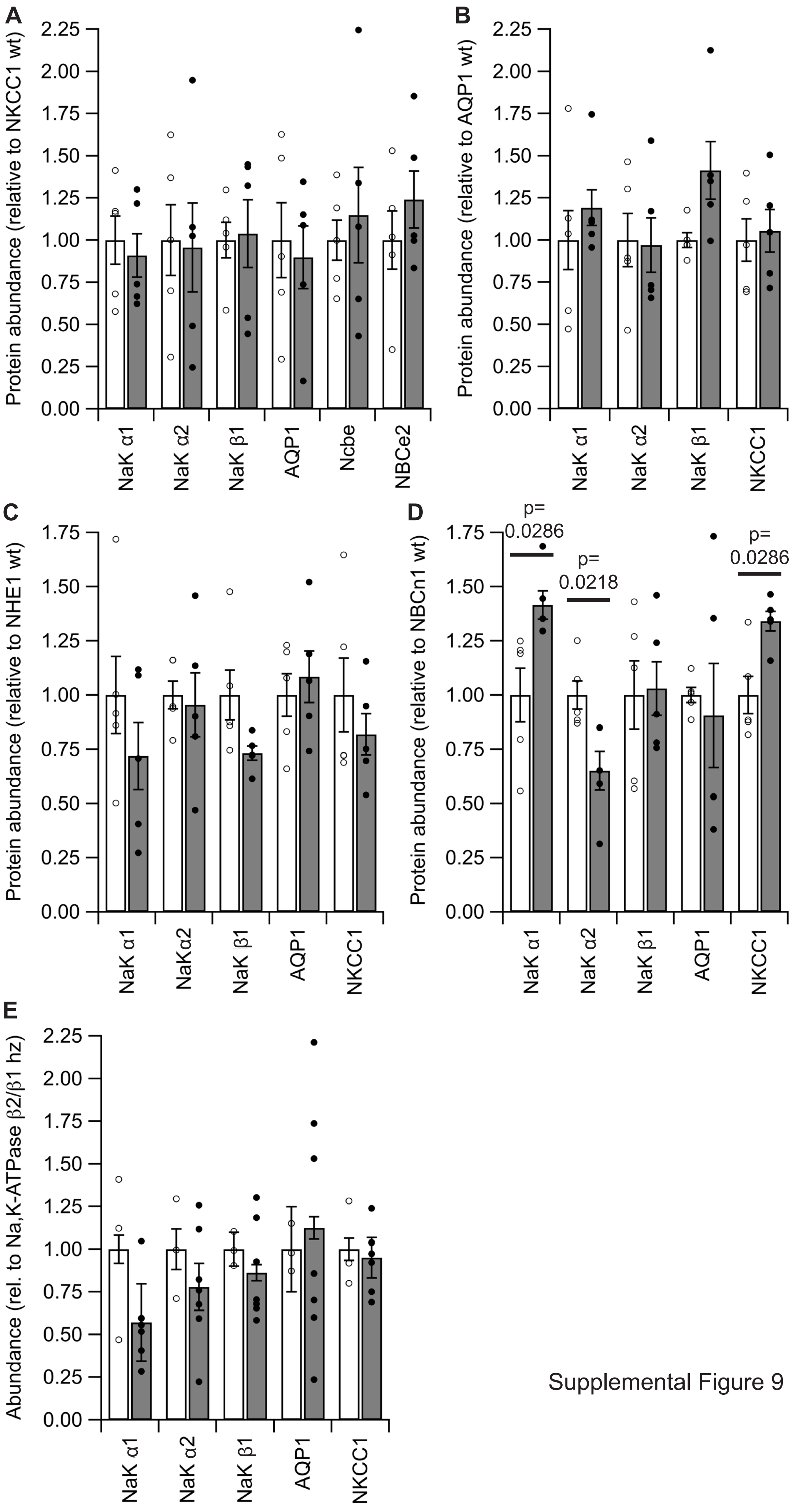

Supplemental Figure 9

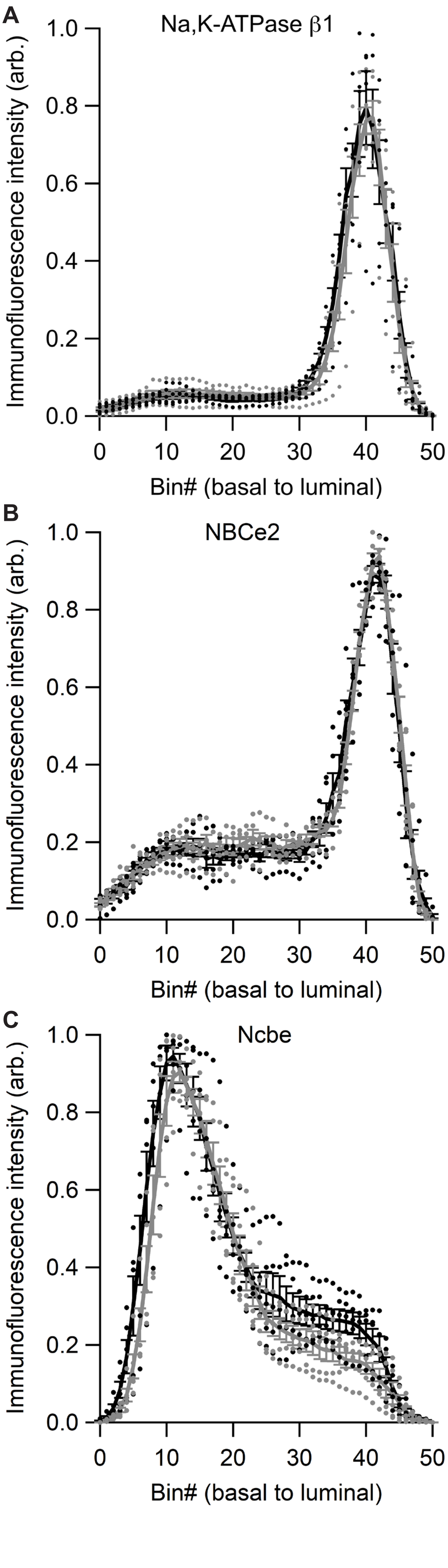

Supplemental Figure 10
